# Using a multiple-delivery-mode training approach to develop local capacity and infrastructure for advanced bioinformatics in Africa

**DOI:** 10.1371/journal.pcbi.1008640

**Published:** 2021-02-25

**Authors:** Verena Ras, Gerrit Botha, Shaun Aron, Katie Lennard, Imane Allali, Shantelle Claassen-Weitz, Kilaza Samson Mwaikono, Dane Kennedy, Jessica R. Holmes, Gloria Rendon, Sumir Panji, Christopher J Fields, Nicola Mulder

**Affiliations:** 1 Computational Biology Division, Department of Integrative Biomedical Sciences, IDM, CIDRI Africa Wellcome Trust Centre, University of Cape Town, Cape Town, South Africa; 2 Department of Biodiversity and Conservation Biology, University of the Western Cape, Bellville, South Africa; 3 Sydney Brenner Institute for Molecular Bioscience, University of the Witwatersrand, Johannesburg, South Africa; 4 Laboratory of Human Pathologies Biology, Department of Biology, Faculty of Sciences, and Genomic Center of Human Pathologies, Faculty of Medicine and Pharmacy, Mohammed V University in Rabat, Rabat, Morocco; 5 Division of Medical Microbiology, Department of Pathology, Faculty of Health Sciences, University of Cape Town, Cape Town, South Africa; 6 Department of Science and Laboratory Technology, Dar es Salaam Institute of Technology, Dar es Salaam, Tanzania; 7 The Inter-University Institute for Data Intensive Astronomy (IDIA), Department of Astronomy, University of Cape Town, Cape Town, South Africa; 8 High Performance Computing in Biology, Roy J. Carver Biotechnology Center, University of Illinois Urbana-Champaign, Urbana and Champaign, Illinois, United States of America; SIB Swiss Institute of Bioinformatics, SWITZERLAND

## Abstract

With more microbiome studies being conducted by African-based research groups, there is an increasing demand for knowledge and skills in the design and analysis of microbiome studies and data. However, high-quality bioinformatics courses are often impeded by differences in computational environments, complicated software stacks, numerous dependencies, and versions of bioinformatics tools along with a lack of local computational infrastructure and expertise. To address this, H3ABioNet developed a 16S rRNA Microbiome Intermediate Bioinformatics Training course, extending its remote classroom model. The course was developed alongside experienced microbiome researchers, bioinformaticians, and systems administrators, who identified key topics to address. Development of containerised workflows has previously been undertaken by H3ABioNet, and Singularity containers were used here to enable the deployment of a standard replicable software stack across different hosting sites. The pilot ran successfully in 2019 across 23 sites registered in 11 African countries, with more than 200 participants formally enrolled and 106 volunteer staff for onsite support. The pulling, running, and testing of the containers, software, and analyses on various clusters were performed prior to the start of the course by hosting classrooms. The containers allowed the replication of analyses and results across all participating classrooms running a cluster and remained available posttraining ensuring analyses could be repeated on real data. Participants thus received the opportunity to analyse their own data, while local staff were trained and supported by experienced experts, increasing local capacity for ongoing research support. This provides a model for delivering topic-specific bioinformatics courses across Africa and other remote/low-resourced regions which overcomes barriers such as inadequate infrastructures, geographical distance, and access to expertise and educational materials.

## Introduction

**Fig 1:**
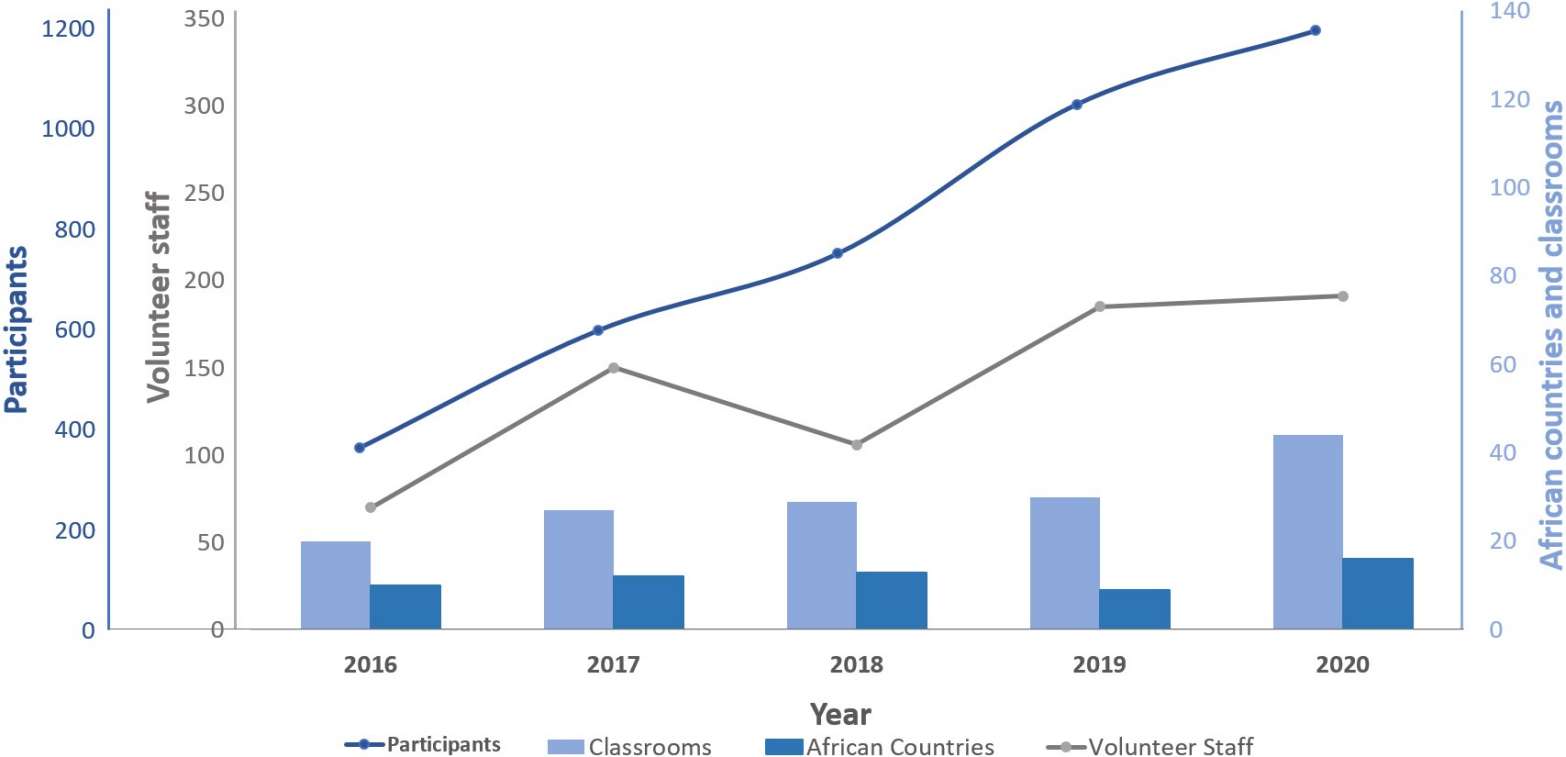
Growth in participation within the H3ABioNet Introduction to Bioinformatics Training Course (IBT) over the period of 2016–2020. Scales have been coloured to match the colours of their associated graphs.

Access to specialist high-quality bioinformatics training in Africa is often hampered by the lack of access to adequate computational infrastructure, locally based trainers with relevant expertise, technical support personnel, and high travel costs [1–4]. In recent years, however, there has been a marked increase in the number of microbiome (and other data-intensive) studies being conducted by African-based research groups, increasing the demand for knowledge in the design and analysis of microbiome data. The Pan African Bioinformatics Network for H3Africa, H3ABioNet, is addressing this demand for training by African scientists through the establishment of topic-specific bioinformatics courses and the development of specific infrastructure coupled with the unique operational model developed to successfully deliver the popular Introduction to Bioinformatics Training Course (IBT) (https://www.h3abionet.org/training/ibt) [5]. The IBT, multiple-delivery-mode training model, uses an innovative combination of distance learning (online lectures and a Learning Management System), Open Educational Resources (OER) (https://en.unesco.org/themes/building-knowledge-societies/oer), and face-to-face learning (local classrooms with support staff) to overcome the shortfalls mentioned above and those highlighted by participants of previous training—particularly, the lack of access to computational resources once the training ends. The IBT course has been delivered annually since 2016, reaching over 3,000 participants across 16 African countries to date (see Fig 1).

The IBT course made use of predominantly online tools to limit local technical challenges however, some of the major impediments to organizing and delivering advanced bioinformatics courses often include differences in computational environments, complicated software stacks, and numerous dependencies and software versions. The development of containerised workflows has previously been undertaken by H3ABioNet and enabled the deployment of a standard replicable software stack without the overhead of installing individual tools, versions, and libraries [6]. This approach has also been successfully used by other organisations offering similar training, such as the European Bioinformatics Institute (e.g., see Course overview) and bioinformatics.ca (see: https://bioinformatics.ca/workshops/). A container can be thought of as an entire runtime environment which includes an application, its dependencies, libraries, configurations, and any other files or binaries required to run the application, within a single package. The key benefit is that this package/container can be decoupled from its environment or operating system (OS) allowing the package/container (and its applications) to be deployed quickly and easily regardless of the target OS or environment. This decoupling has the added benefit of “separating” tasks. Developers can focus on the design of the applications and its libraries, dependencies, etc., while an IT team can focus on deployment and management. The use of containers thus appeared to be the optimal approach for delivering advanced bioinformatics training using the IBT remote classroom model.

**Fig 2:**
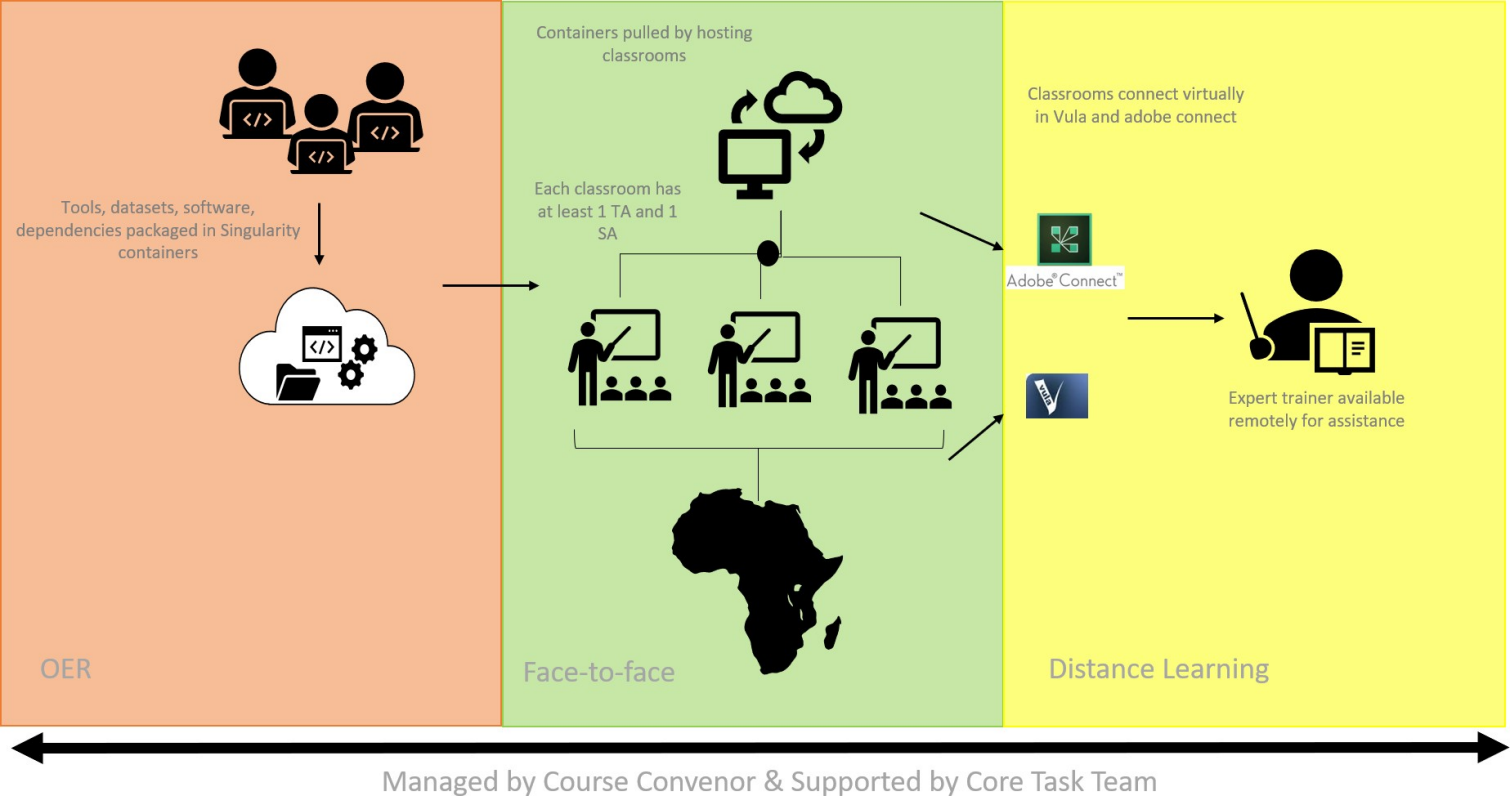
Diagrammatic depiction of the multiple-delivery-mode training model as it was applied within the H3ABioNet 16S rRNA Intermediate Bioinformatics Training course during 2019. The figure illustrates the 3 major modalities employed within the course, which are OER (Open Educational Resources), face-to-face learning, and online/distance-based learning. We also depict the staffing structure.

A task force within the Training and Education Work Package of the H3ABioNet consortium has developed the “H3ABioNet 16S rRNA Microbiome Intermediate Bioinformatics Training” course (16S course), with the hope of testing the use of a containerised approach for providing advanced bioinformatics training, delivered using the multiple-delivery-mode training model (Fig 2 provides an overview of the model as it was applied within this course). The success of this approach would ensure that participating host sites develop capacity and have access to local microbiome data analysis resources and expertise posttraining. This model could also allow advanced bioinformatics training to reach more remote regions not only in Africa but in other low-resourced countries across the world too.

### Course development

The course was developed over a 6-month period and involved in-depth consultation with both subject experts and experienced bioinformaticians comfortable with developing containers. Due to the underlying inequalities in infrastructure across institutions in Africa, the development of the content and infrastructure became highly challenging, and these inequalities needed to be considered in every approach taken by the course developers. The main developmental and operational steps have been outlined in the sections below.

### Competency-based curriculum development

The H3ABioNet 16S course was developed together with experienced microbiome researchers who identified key topics ranging from designing microbiome studies to more practical topics such as introductory high-performance computing (HPC), microbiome analysis pipelines, and downstream analyses conducted in R. For each of the identified topics, roughly 5 learning outcomes were developed and mapped to bioinformatics core competencies (as developed and suggested by the International Society for Computational Biology (ISCB)) [7,8]. The ISCB core competencies were originally designed with “Personas” (“Bioinformatics User,” “Bioinformatics Scientist,” and “Bioinformatics Engineer”) in mind, with different competencies often applying to different Personas and at various levels of complexities. A Bioinformatics User (e.g., a molecular/life scientist) would, for example, require different competencies to a Bioinformatics Engineer (e.g., programmer/developer) or require the same competencies but at much different levels of complexity. Bloom’s taxonomy provides a hierarchical approach to learning, providing various (defined) levels within the pedagogy of the learning path. Once we determined our Persona (for our purposes a Bioinformatics Scientist), the application of Bloom’s taxonomy allowed us to determine which competencies needed to be developed for this Persona (as per ISCB’s recommendations) and to what level/depth for the training and Persona. Competencies were then further mapped to Knowledge, Skills, and Attitudes (KSAs) resulting in the development of 6 core modules outlined in Table 1 below.

**Table 1:** Mapping of bioinformatics core competencies to individual modules taught as part of the H3ABioNet 16S rRNA Microbiome Intermediate Bioinformatics Training course. The identified Bloom’s Taxonomy for each competency is also shown. Detailed competency mapping was performed by module trainers to drive content development within the course.

Persona: Bioinformatics Scientist		
Module	Competency/ies	Bloom’s Taxonomy
**Introduction to the Linux command line/Introduction to R**	F—Bioinformatics tools and resources and their usage	Application
	G—Fundamentals of computer science systems	Application
	J—Scripting and programming appropriate to the discipline	Application
**Introduction to the microbiome and study design—why 16S rRNA?**	A—General biology	Comprehension
	B—Depth in at least 1 area of biology	Comprehension
	D—Details of the scientific discovery process and of the role of bioinformatics in it	Comprehension
**Sample collection, extraction, and library prep for 16S NGS analyses**	A—General biology	Comprehension
	B—Depth in at least 1 area of biology	Comprehension
	C—Biological data generation technologies	Comprehension
**16S rRNA sequencing bioinformatics pipeline: the theory**	A—General biology	Comprehension
	C—Biological data generation technologies	Comprehension
	E—Statistical research methods in the context of molecular biology, genomics, medical, and population genetics research	Comprehension
	F—Bioinformatics tools and resources and their usage	Comprehension
	H—Computing requirements appropriate to solve a given scientific problem	Knowledge
	J—Scripting and programming appropriate to the discipline	Knowledge
**16S rRNA analysis pipeline—QC, OTU picking/ASV analysis, classification, alignment, Usearch, and QIIME**	F—Bioinformatics tools and resources and their usage	Analysis
	H—Computing requirements appropriate to solve a given scientific problem	Analysis
	J—Scripting and programming appropriate to the discipline	Analysis
**Downstream analysis in R—Phyloseq, NMF**	E—Statistical research methods in the context of molecular biology, genomics, medical, and population genetics research	Analysis
	F—Bioinformatics tools and resources and their usage	Analysis
	H—Computing requirements appropriate to solve a given scientific problem	Application
	J—Scripting and programming appropriate to the discipline	Analysis

### Development and deployment of computational infrastructure

One of the main aims of the course was to provide a realistic computational environment for the analysis and interpretation of results, which participants were required to familiarise themselves with. The minimum infrastructural criteria that institutions needed to meet thus included: user access to a Unix environment using the Secure Shell protocol on an Ubuntu compute cluster or desktop machines (versions 16.04 or 18.04) with the minimum specifications of 16 GB of RAM per core; access to a shared 100 GB storage location for the reference sequence databases and 200 GB storage per user for the processing of files during the course; installation of Singularity (version 3.6.2) and *overlayfs* enabled; Nextflow (version 20.07.1) and Java (version 1.8 or higher); and hosting of an R-Studio (version 1.2.5042) Singularity container with the required R (version 3.6.3) analysis and visualisation packages installed with the ability for users to interactively log into a compute node to execute the container (https://github.com/ilifu/bioinformatics/blob/master/containers/R/RStudio/bionic-R3.6.3-RStudio1.2.5042-bio.def) for further analysis.

Each hosting Institution had a Systems Administrator (SA) available for the core team to interact with to help them set up the course containers and software on their compute environments and, together with the Teaching Assistants (TAs), provide support to their students for the duration of the course. Prior to the course start date, the core team organised interactive sessions with a total of 20 SAs to ensure that the required computational environment was provisioned correctly by testing the pulling and running of the containers, workflows, and datasets, and troubleshooting to rectify any errors.

H3ABioNet previously developed containerised reproducible workflows for heterogeneous computational environments encountered within Africa [6]. An initial 16S rRNA Microbiome workflow was redeveloped to version 2 using Nextflow (version 20.07.1) and nf-core design principles [9] consisting of 2 Docker containers, one for NGS QC (version 17.09.0-ce) and the second Docker container (version 19.03.11) incorporating the DADA2 pipeline (version 1.14.0) and its various dependencies that are built, tested, and hosted on Quay.IO (docker pull quay.io/h3abionet_org/h3a16s-fastqc; docker pull quay.io/h3abionet_org/16s-rdna-dada2-pipeline). The Docker containers are executed as Singularity containers to accommodate typical compute cluster and HPC environments and utilisation of schedulers when using a shared compute resource with multiple users for NGS analysis. Version 2 of the TADA (Targeted Amplicon Diversity Analysis) workflow includes support for alternative 16S regions and non-16S amplicons (ITS, mitochondrial CO1, custom amplicons) and alternate workflows supporting longer amplicon analyses such as full-length 16S rRNA data (PacBio, Loop Genomics). Functionality was included to generate output formats for use in other tools including BIOM, QIIME2 (QZA), simple tab-delimited text output, R data files (RDS) for phyloseq analysis, and preliminary support for data generation from 2-colour Illumina sequencing (NovaSeq, NextSeq). The TADA workflow has continuous integration (CI) implemented using Travis-CI (a platform that tests code on every commit to the repository by running the newly generated workflow code against test data) and extensive documentation detailing the parameters available and how to run the workflow.

**Fig 3:**
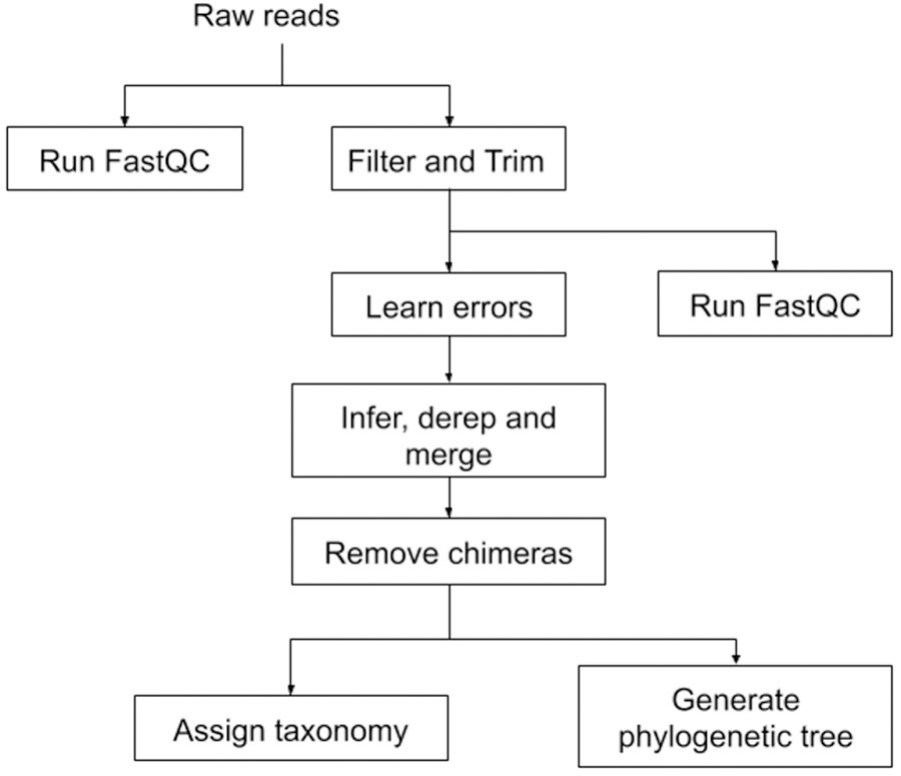
Simplified schematic representation of the 16S rRNA analysis workflow. The complete TADA workflow is accessible on GitHub: https://github.com/h3abionet/TADA.

The TADA workflow utilises the container with FastQC [10] (version 0.11.5) for checking raw read quality and MultiQC [11] (version 1.6) for aggregating and generating quality control reports for the input dataset. Read trimming and filtering is performed primarily in R by DADA2 [12] with another round of QC reports being generated for comparison. Sequence error rates for the forward and reverse reads are estimated using the DADA2 learnErrors method, the sample data are then pooled, denoised, dereplicated, and merged. Chimeras are removed and amplicon sequence variants (ASVs) identified. Taxonomic rank assignment is further executed in R using the DADA2 RDP Classifier implementation and the Silva database (version 11.5) [13], with multiple sequence alignment performed by DECIPHER (version 2.14.0) [14] and phylogenetic analysis performed using phangorn (version 2.5.5) [15]. The resulting phylogenetic tree is midpoint-rooted using ape (version 5.3) [16], and the BIOM [17] file produced is used in the course for further downstream analysis using the R-Studio container (see Fig 3 for a simplified schematic of the workflow). The workflow is open-source (MIT licence) and available on GitHub: https://github.com/h3abionet/TADA.

#### Dataset description

The dataset used for the training consisted of paired-end Illumina short read sequences containing 15 dog stool samples and required a total 2.4 GB of disk space. The dataset was obtained from collaborators at the University of Illinois Urbana-Champaign and chosen because the dataset was of nonhuman origin so minimal ethical considerations were needed. Additionally, the data showed a pattern of diversity among the samples that linked well to some observations, therefore demonstrated additional promise as training data.

The reads were sequenced from the V3 to V4 region, and 300 bp read pairs were generated. S1 Table contains the metadata associated with the dog stool samples. Three dogs were treated with an increased percentage of a compound in their diet: 5 different treatments (0 to 4, representing an increased percentage of a compound in their diet). The workflow typically takes approximately 28 minutes and 1.7 CPU hours to complete on 4 samples used for testing the computational environment.

### The multiple-delivery-mode learning model employed by this course

The course organisers followed the IBT [5] operational model quite closely; however, some major deviations from the IBT model were required due to the more complex practical components of this course and have been summarised in Table 2.

**Table 2:** Comparison of the multiple-delivery-mode training model employed by H3ABioNet’s Introduction to Bioinformatics (IBT) course and the 16s rRNA Microbiome Intermediate Bioinformatics Training course (16S). The table provides a short description of the major components of the model employed by each course, highlighting any differences between the two (deviations are indicated by an asterisk (*)).

		IBT	16S
**Face to face**	*Classrooms	Formal application and selection process. Required to meet some basic criteria like access to a projector and a low specification computer facility. Core team selected final classrooms based on application information	Formal application and selection process. Required advanced facilities with access to a cluster and classrooms thoroughly vetted by core team. Hosts required to perform full testing and provide outputs of test analyses before approval
	*Staff	Course required TAs with basic knowledge. TAs could handle duties of the SA	Required skilled TAs and a highly experienced SA. Course could not continue without the SA
	*Participant applications and selections	Formal application and selection process. Participants required to meet basic criteria. Local classrooms performed final selection	Formal application and selection process. Participants required to have advanced knowledge in either R or Unix. Local classrooms performed final selection
	**Contact sessions**	Biweekly, 4 hours per day for 3 months	Biweekly, 4 hours per day for 2 months
**Distance learning**	**Lectures**	Prerecorded lectures by expert trainers made available ahead of modules	Prerecorded lectures by expert trainers made available ahead of modules
	**Live sessions**	Biweekly live Q&A/discussion sessions with module trainer, facilitated using Adobe Connect or Zoom	Biweekly live Q&A/discussion sessions with module trainer, facilitated using Adobe Connect or Zoom
	**Vula**	LMS (http://vula.uct.ac.za) used to administer structured forums, assignments, tests, and evaluations. Vula was also used to provide lecture materials	LMS (http://vula.uct.ac.za) used to administer structured forums, assignments, tests, and evaluations. Vula was also used to provide lecture materials and R scripts
**OER**	**Vula**	Content remains available indefinitely after course end and are under a creative commons license allowing anyone to reuse it with the proper credit	Content remains available indefinitely after course end and are under a creative commons license allowing anyone to reuse it with the proper credit
	**YouTube**	All lecture videos made available directly on Vula and via a publicly accessible YouTube playlist	All lecture videos made available directly on Vula and via a publicly accessible YouTube playlist
	*Datasets	Fasta files and vcfs made available for simple plug and play style analyses	Required datasets shared via Vula and via GitHub repositories for advanced computational analyses
	*Code	Some basic code shared as part of lecture slides	Advanced code shared on Vula and via GitHub repositories, e.g., https://iallali.github.io/DADA2_pipeline/16SrRNA_DADA2_pipeline.html
	*Capacity development	Mostly online tools used so tools remain accessible after course ends; requires no local maintenance	Infrastructure remains at institution to be used in real analyses in the future; requires ongoing maintenance

LMS, Learning Management System; OER, Open Educational Resources; SA, System Administrator; TA, Teaching Assistant.

### The Pilot: Successes, challenges, and future improvements

The pilot of the course ran successfully in 2019 at 23 hosting sites registered across 11 African countries (more information can be found here: https://www.h3abionet.org/training/int-bt). More classrooms expressed interest in the pilot than was initially anticipated, resulting in 264 participants being formally enrolled in the course. There were also 106 volunteer staff available for onsite support across the 23 classrooms. In order to successfully complete the course and obtain the course letter of completion, participants needed to obtain an average of 60% across all module tests, submit at least 90% of all practical assignments, and attend at least 90% of all contact sessions within their local classrooms. Course feedback surveys were conducted throughout the course to continually assess the relevance and quality of content and lectures and to gauge participant confidence in utilising the various tools.

**Key successes: Participants–**146 participants met the requirements to pass the course and obtained the course letter of completion. At most hosting sites, classrooms allowed a period of access to containers postcourse to allow participants to analyse their own data, while others gained the confidence to apply for formal access to local compute facilities and performed analyses there.

**Containers**–containers worked as expected across all sites using a compute cluster. Containers remained at hosting sites for further use (and to upscale as needed).

**Reproducibility–**Nextflow raw outputs were requested from all sites (and included results from random students) to test reproducibility and all these results were identical. This supported the conclusion that containers could allow results to be replicated by any site with adequate infrastructure.

**Availability of resources postcourse**–this approach ensured tools and workflows were set up and accessible locally and are available to use on larger datasets after the course concludes, strengthening the local institutional capacity.

**Fig 4:**
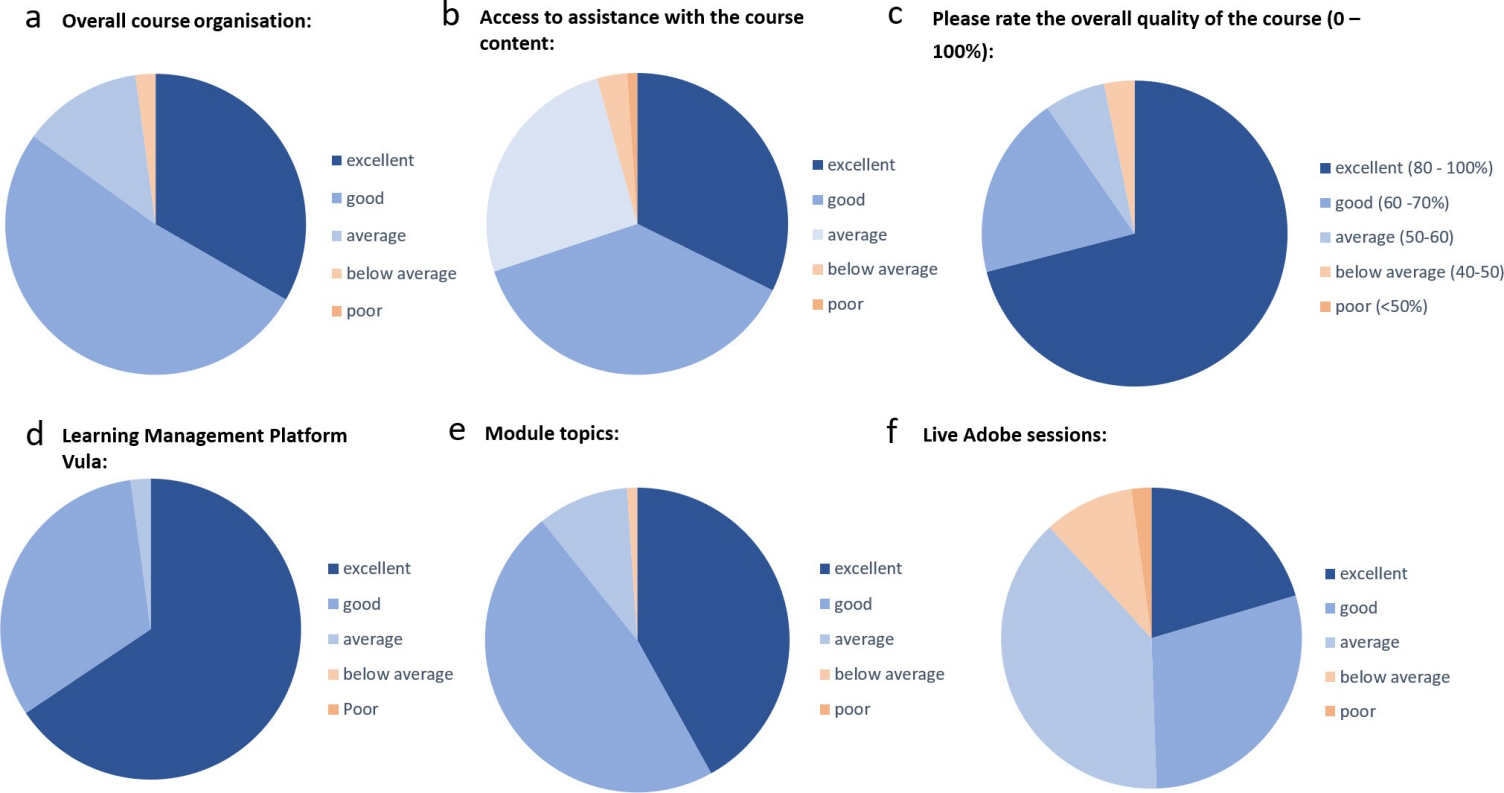
Responses from feedback surveys for randomly selected questions to illustrate participant satisfaction with the H3ABioNet 16S rRNA Microbiome Intermediate Bioinformatics Training course (https://www.h3abionet.org/training/int-bt). Surveys were all administered within the learning management system Vula (http://vula.uct.ac.za). These summaries were compiled from a total of 93 responses across all questions.

**Participant feedback during evaluations–**feedback was generally positive and some of the responses have been highlighted in Fig 4. Based on evaluations, the quality of the course was voted in the 80th percentile or higher by up to 74% of respondents and 100% of participants indicated they would recommend the course to someone else.

**Challenges and future improvements: Classroom resources**–one of the main challenges with this approach is the computational requirements for the hosting site. Some classrooms could not participate as they could not meet classroom specifications and the organising team occasionally needed to host additional participants.

**Local expertise**–for the course to run effectively, a dedicated, experienced SA was required. These skills were not always immediately available at all sites, and, on occasion, SAs had to move between hosting sites to provide support. Like the IBT course, the success of each classroom was driven by the local staff at each site. The course had 2 dedicated days of staff (facilitation) training, and the core technical team delivered extensive training on the pulling and running of containers during both group and one-on-one sessions prior to the course. The consensus from informal evaluations was that this was not enough time to become comfortable with the content and that the training needed to be more exhaustive. We are aiming to extend staff training for future iterations.

**Module duration**–several participants indicated that the introductory modules were too short and suggested these modules be increased in length. The core team is reviewing this for the upcoming iterations. The inclusion of some compulsory prerequisite online courses might be used in future iterations of the course to ensure that the participants have a similar foundational knowledge in specific areas such as Linux and R.

**Contact sessions**–a few participants indicated that engagement between classrooms during live virtual sessions was low. This could be encouraged by the core team and trainers who are looking at designing more interactive contact session plans.

**Ongoing trainee tracking**–H3ABioNet performs a biannual training survey which collects ongoing information on the progression of their trainees. The survey collects information around whether attending a training has resulted in any publications or outputs and tracks whether the training assisted in the career progression of the trainees in any way. All participants participating in this course have been added to this survey for ongoing monitoring.

## Conclusions and future implications

In 2020 when the Coronavirus Disease 2019 (COVID-19) pandemic hit, training activities were hugely impacted by lockdowns and social distancing measures, and many institutions struggled to continue their planned activities with all cancelling face-to-face training and workshops. Although a need for distance-based training already existed within Africa (and the world), the pandemic further emphasised the need for increased online training and blended solutions, particularly within low-resourced settings. The pilot H3ABioNet 16S course ran successfully in 2019 and provides a model for delivering topic-specific bioinformatics courses across the African continent, which overcomes some of the barriers caused by unequal infrastructures, geographical location, access to expertise, and high-quality educational materials. In the face of a pandemic, such as COVID-19, it also provides a relatively fast solution to delivering advanced remote training without compromising on quality. Although H3ABioNet has focused on the specific needs of African institutions, the model is adoptable in any setting (low and well-resourced). The greatest value of this model is its scalability and generalizability to a wide range of topics, not confined to bioinformatics alone.

The local capacity built during the course and availability of containers postcourse coupled with the use of OER ensure institutions can continue to train additional researchers even if the course is no longer available. The model is currently being used to develop further intermediate courses within H3ABioNet. H3ABioNet has also recently partnered with international training providers to assist them in implementing the model for their purposes. Finally, based on the COVID-19 restrictions, we adapted the course to run completely online for the 2020 iteration. This highlights the flexibility of the model being able to go fully online or fully face-to-face as the need arises.

## Supporting information

S1 Table Training dataset metadata. Contains information on sample collection, dog treatment, and read counts.
(DOCX)

## References

[1] Ojo OO, Omabe M. Incorporating bioinformatics into biological science education in Nigeria: Prospects and challenges. Infect Genet Evol. 2011;11. 10.1016/j.meegid.2010.11.015 21145989

[2] Tastan Bishop O, Adebiyi EF, Alzohairy AM, Everett D, Ghedira K, Ghouila A, et al. Bioinformatics Education—Perspectives and Challenges out of Africa. Brief Bioinform. 2015;16. 10.1093/bib/bbu022 24990350 PMC4364068

[3] Mulder NJ, Adebiyi E, Alami R, Benkahla A, Brandful J, Doumbia S, et al. H3ABioNet, a sustainable pan-African bioinformatics network for human heredity and health in Africa. Genome Res. 2016;26. 10.1101/gr.196295.115 26627985 PMC4728379

[4] Karikari TK. Bioinformatics in Africa: The Rise of Ghana? PLoS Comput Biol. 2015;11. 10.1371/journal.pcbi.1004308 26378921 PMC4574930

[5] Gurwitz KT, Aron S, Panji S, Maslamoney S, Fernandes PL, Judge DP, et al. Designing a course model for distance-based online bioinformatics training in Africa: The H3ABioNet experience. PLoS Comput Biol. 2017;13. 10.1371/journal.pcbi.1005715 28981516 PMC5628786

[6] Baichoo S, Souilmi Y, Panji S, Botha G, Meintjes A, Hazelhurst S, et al. Developing reproducible bioinformatics analysis workflows for heterogeneous computing environments to support African genomics. BMC Bioinformatics. 2018;19. 10.1186/s12859-018-2446-1 30486782 PMC6264621

[7] Welch L, Lewitter F, Schwartz R, Brooksbank C, Radivojac P, Gaeta B, et al. Bioinformatics Curriculum Guidelines: Toward a Definition of Core Competencies. PLoS Comput Biol. 2014;10. 10.1371/journal.pcbi.1003496 24603430 PMC3945096

[8] Mulder N, Schwartz R, Brazas MD, Brooksbank C, Gaeta B, Morgan SL, et al. The development and application of bioinformatics core competencies to improve bioinformatics training and education. PLoS Comput Biol. 2018;14. 10.1371/journal.pcbi.1005772 29390004 PMC5794068

[9] Ewels PA, Peltzer A, Fillinger S, Patel H, Alneberg J, Wilm A, et al. The nf-core framework for community-curated bioinformatics pipelines. Nat Biotechnol. 2020;38. 10.1038/s41587-020-0439-x 32055031

[10] Andrews S. FastQC: a quality control tool for high throughput sequence data. 2010 [cited 5 May 2012]. Available from: http://www.bioinformatics.babraham.ac.uk/projects/fastqc

[11] Ewels P, Magnusson M, Lundin S, Käller M. MultiQC: summarize analysis results for multiple tools and samples in a single report. Bioinformatics. 2016;32. 10.1093/bioinformatics/btw354 27312411 PMC5039924

[12] Callahan BJ, McMurdie PJ, Rosen MJ, Han AW, Johnson AJA, Holmes SP. DADA2: High-resolution sample inference from Illumina amplicon data. Nat Methods. 2016;13. 10.1038/nmeth.3869 27214047 PMC4927377

[13] Glöckner FO, Yilmaz P, Quast C, Gerken J, Beccati A, Ciuprina A, et al. 25 years of serving the community with ribosomal RNA gene reference databases and tools. J Biotechnol. Elsevier B.V.; 2017. pp. 169–176. 10.1016/j.jbiotec.2017.06.1198 28648396

[14] Wright ES. RNAconTest: comparing tools for noncoding RNA multiple sequence alignment based on structural consistency. RNA. 2020;26. 10.1261/rna.073015.119 32005745 PMC7161358

[15] Schliep K, Potts AJ, Morrison DA, Grimm GW. Intertwining phylogenetic trees and networks. Methods Ecol Evol. 2017;8. 10.1111/2041-210X.12760

[16] Paradis E. Schliep K. ape 5.0: an environment for modern phylogenetics and evolutionary analyses in R. Bioinformatics. 2019;35. 10.1093/bioinformatics/bty633 30016406

[17] McDonald D, Clemente JC, Kuczynski J, Rideout JR, Stombaugh J, Wendel D, et al. The Biological Observation Matrix (BIOM) format or: how I learned to stop worrying and love the ome-ome. GigaScience. 2012;1. 10.1186/2047-217X-1-7 23587224 PMC3626512

