# Supplementary material for: Using a multiple-delivery-mode training approach to develop local capacity and infrastructure for advanced bioinformatics in Africa
Source: PLoS Comput Biol. 2021 Feb 25;17(2):e1008640. doi: 10.1371/journal.pcbi.1008640 (PMC7906323; doi:10.1371/journal.pcbi.1008640)
Supplement: S1 Table — Contains information on sample collection, dog treatment, and read counts. (DOCX) [file pcbi.1008640.s001.docx]

| **Sample** | **Dog** | **Treatment** | **Read Counts r1** | **Read Counts r2** |
| --- | --- | --- | --- | --- |
| Dog1 | B | 2 | 118343 | 118343 |
| Dog2 | G | 3 | 108679 | 108679 |
| Dog3 | K | 3 | 101482 | 101482 |
| Dog8 | B | 4 | 108731 | 108731 |
| Dog9 | G | 0 | 109500 | 109500 |
| Dog10 | K | 4 | 79342 | 79342 |
| Dog15 | B | 1 | 131483 | 131483 |
| Dog16 | G | 4 | 114424 | 114424 |
| Dog17 | K | 0 | 99610 | 99610 |
| Dog22 | B | 3 | 145029 | 145029 |
| Dog23 | G | 1 | 193158 | 193158 |
| Dog24 | K | 2 | 162487 | 162487 |
| Dog29 | B | 0 | 122776 | 122776 |
| Dog30 | G | 2 | 137315 | 137315 |
| Dog31 | K | 1 | 150613 | 150613 |
